# Solitary Splenic Tuberculosis in an Immunocompetent Child: A Case Report

**DOI:** 10.7759/cureus.5210

**Published:** 2019-07-23

**Authors:** Assadullah Metlo, SM Ismail Shah, Aiman Rehan, Syed Hamza Bin Waqar, Rabbia Siddiqi

**Affiliations:** 1 Pediatrics, Civil Hospital, Karachi, PAK; 2 Internal Medicine, Ziauddin Medical College, Karachi, PAK; 3 Internal Medicine, Dow University of Health Sciences, Karachi, PAK; 4 Internal Medicine, Civil Hospital Karachi, Dow University of Health Sciences, Karachi, PAK

**Keywords:** tuberculosis, spleen, immunocompetent, child, splenic abscess

## Abstract

Tuberculosis (TB) is a lethal infectious disease that still remains a major threat in developing countries. Solitary splenic tuberculosis is a rare entity and there have been very few cases of it reported in literature. It is mostly encountered in patients who have an immunocompromised state. It may occur with a myriad of non-specific presentations, making it complex to diagnose. Here, we report a case of an eight-year-old female, immunocompetent, who had complaints of fever, abdominal pain and chronic diarrhea. Laboratory data failed to provide any information about the final diagnosis. On physical examination, splenomegaly was present. Imaging studies were conducted with an abdominal ultrasound showcasing mild ascites, splenomegaly, with a homogeneous echo pattern and no focal mass. Computed tomography (CT) of the abdomen showed two hypodense areas in the subcapsular region of the spleen and extending into the capsule, suggestive of a tuberculous abscess with mesenteric lymphadenopathy. The diagnosis was further corroborated when the patient showed remarkable improvement on anti-tuberculous therapy. This is a very uncommon phenomenon, especially in an immunocompetent patient and hence, it is very important to keep this on the list of differentials especially in an area where TB is endemic.

## Introduction

Tuberculosis (TB) is a worrisome clinical entity especially in developing countries and has a variety of presentations. It is usually classified into two categories: pulmonary and extra-pulmonary. Extra-pulmonary TB accounts for 15-20% of all cases, commonly involving the central nervous system, pleura, lymphatic system, bones and joints [1]. However, virtually any organ can be involved. Abdominal tuberculosis may involve the gastrointestinal tract, peritoneum, mesenteric lymph nodes, or genitourinary tract. Usually, splenic TB only manifests as a part of miliary TB. However, cases of isolated splenic tuberculosis are an extremely rare presentation [2]. In recent years, childhood TB has received more attention with emphasis on the improvement of TB diagnostic techniques in children. Furthermore, in 25% to 35% of children, TB is extra-pulmonary, most commonly being acquired from the community [3, 4]. Here, we present a case of an eight-year-old immunocompetent child with primary splenic TB.

## Case presentation

An eight-year-old Pakistani female child, unvaccinated, and from a poor socio-economic background, presented to the emergency department with complaints of fever and intermittent abdominal pain for one year. The fever was low-grade (100°F on examination) and was not associated with rigors, chills or night sweats. The patient also had generalized abdominal pain, which occasionally subsided after passing stools. She also had a four-year history of chronic diarrhea attributed to malabsorption. Her stools were yellow green in color, bulky, difficult to flush, greasy and foul smelling, but did not contain any blood. The patient had about three to five episodes of diarrhea per day. There was no history of constipation or delayed passage of meconium at birth.

**Figure 1:**
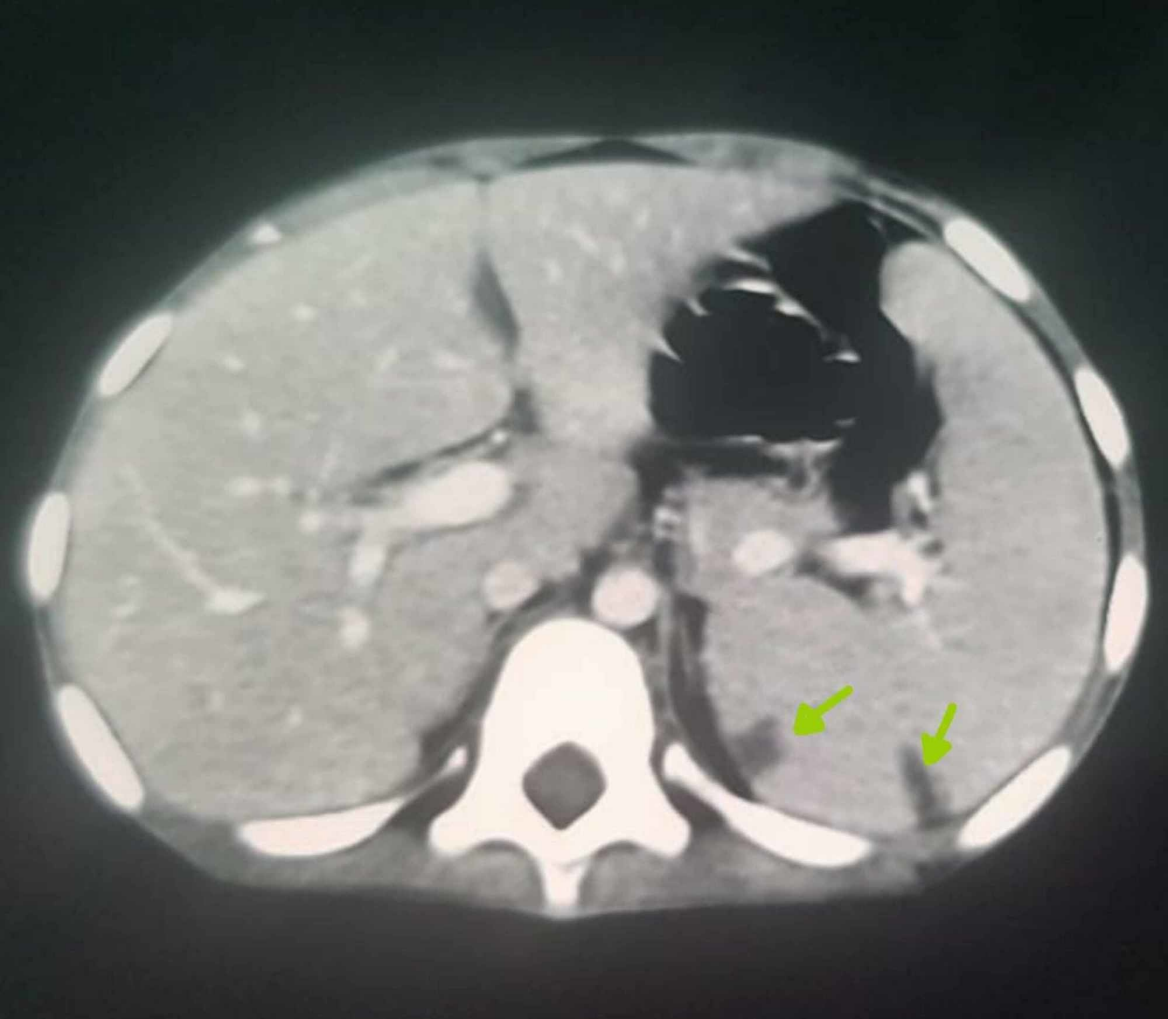
Axial computed tomography (CT) of abdomen with contrast. Axial CT of abdomen with contrast showing two subcapsular hypodense areas (shown by the arrows) signifying abscesses.

Physical examination showed a pale looking, conscious and cooperative child, with a thin built. The patient’s height and weight were below normal for age: 106 cm (below 10th centile), and 16 kg (below 10th centile), respectively. Her vitals were within normal limits. Abdominal exam revealed a soft, non-tender and mildly distended abdomen with the spleen palpable 2 cm below the left costal margin. The rest of the physical exam was within normal limits. Complete blood count showed microcytic anemia with hemoglobin of 8 g/dl, mean corpuscular volume 65.7 fl, mean corpuscular hemoglobin 15.9 pg and mean corpuscular hemoglobin concentration 25.1 g/dl. Total leucocyte count was 20.3 x 103 cells/cubic millimeter, lymphocytes 19%, neutrophils 77%, eosinophils 5% and monocytes were 4%. Her erythrocyte sedimentation rate (ESR) was elevated with 15/hr. Stool detailed report sample was negative for mucus, worms, blood, ova, cysts and parasites, however, there were 2-4/hpf of pus cells. Fat globules and food remnants were also present. Tissue transglutaminase IgA and IgG were normal, ruling out celiac disease. Fecal antigens for giardiasis were negative. Abdominal ultrasound revealed mild ascites, splenomegaly, with a homogeneous echo pattern and no focal mass. Figure 1 shows a computed tomography (CT) of the abdomen with two hypodense areas seen in the subcapsular region of spleen and extending into the capsule. One of these areas measured 1.1 x 0.9 cm, and the other 1.3 x 0.6 cm. The overall appearance was, hence, suggestive of a tuberculous abscess in the subcapsular region with mesenteric lymphadenopathy.

Splenic biopsy was not performed due to a potential risk of rupture. There was no other focus of tuberculosis in the lungs, as confirmed by the chest X-ray. Her Mantoux test, gene-Xpert, and acid-fast bacilli (AFB) culture of sputum, all came out negative. Regardless, due to clinical suspicion of TB, the patient was started with quadruple anti-TB therapy (ATT) for 12 months which improved her symptoms within two weeks. Her diarrhea also resolved. At the last follow-up, she had completed four months of ATT treatment, was afebrile and gaining weight. Informed consent was taken from the patients’ parents for publishing this case.

## Discussion

Primary tuberculosis of the spleen is an unusual presentation. Most such reported cases occur in HIV-infected or otherwise immunocompromised patients; splenic lesions are only sporadically found in immunocompetent individuals. A suspicion for isolated tuberculosis of the spleen should be made with fever of unknown origin and abdominal pain, especially in patients from endemic areas [5]. The symptoms for splenic TB are usually non-specific and deceptive, including fever, diarrhea, abdominal pain, weight loss and anorexia. It can be asymptomatic as well [6]. Anemia is also seen in some cases, either as microcytic or normocytic, and elevated ESR may also be an indicator [7, 8]. The diagnosis may be complicated by the absence of tuberculous lesions in other organs, especially in the lungs, from where there is a possibility of hematogenous spread to the spleen (miliary tuberculosis). In our case, the patient also gave a negative response to the Mantoux test, and gene-Xpert and AFB stain and culture were also unhelpful in the diagnosis. She was also immunocompetent, which is rarely associated with primary splenic tuberculosis [9]. However, there are no strict patterns of this disease, and variant presentations are possible, whether it is in the form of a primary tuberculous lesion of the spleen, or miliary tuberculosis excluding the lungs [10, 11].

Abdominal tuberculosis manifests as tubercular lymphadenopathy, peritoneal tuberculosis, visceral tuberculosis and gastrointestinal tuberculosis [12, 13]. Our case was of a tuberculous abscess, a form of visceral tuberculosis affecting the liver, genitourinary system and spleen etc.; however, there was also mesenteric lymphadenopathy, which makes it a hybridized case of tubercular lymphadenopathy with visceral tuberculosis. To our knowledge, only one such case of a child has been reported [14]. Splenic abscess is a rare presentation, which is usually found in immunodeficiency conditions, and due to hematogenous bacterial dissemination or trauma. It is one of the five patho-morphological classifications for splenic tuberculosis. The other four include miliary tuberculosis, nodular tuberculosis, calcific tuberculosis, and mixed type tuberculosis [15]. In this case, the patient had a history of intermittent fever for one year, which along with radiological findings and other symptoms, led to the discovery of a splenic abscess. Imaging studies such as ultrasound and CT scan have proved highly sensitive to identify splenic abscesses, there are no characteristic findings that are only specific for TB [16]. The radiologic appearance of tuberculous lesions is divided into micro nodular and macro nodular forms. Micro nodular are usually small, multiple nodules which calcify in chronic conditions, while macro nodular are rare, single, large and tumor-like lesions [17]. The gold standard method of diagnosis involves performing a splenectomy and taking biopsies. For histopathologic diagnosis, both fine needle aspiration cytology (FNAC) and core needle biopsy (CNB) are debated in literature, FNAC having a lower risk of bleeding but CNB giving a higher biopsy yield [18]. Laparoscopic biopsy has also been considered [19]. In our case, this was not performed because of a risk of rupture.

Similar to that of pulmonary TB, splenic lesions are also treated with regular and proper administration of oral anti-tuberculous drugs. Sometimes, this is augmented with a splenectomy. In our case, the patient showed improvement with oral mycobacterial therapy.

## Conclusions

According to the Global Tuberculosis Report 2017 by World Health Organization, the top five high burden countries for TB, with 56% of estimated cases, are India, Indonesia, China, the Philippines and Pakistan. TB is the 9th leading cause of death worldwide. Vaccination against TB provides protection to children, even against severe forms of the disease, such as miliary tuberculosis and tuberculous meningitis. Our patient was unvaccinated, which was probably a major risk factor for her disease. In conclusion, we describe a highly atypical presentation of splenic TB, highlighting the necessity of holding a high index of clinical suspicion for TB in endemic areas, even in immunocompetent individuals presenting with non-specific symptoms of TB.
